# Theoretical Study of Molybdenum Separation from Molybdate Assisted by a Terahertz Laser

**DOI:** 10.3390/molecules29143348

**Published:** 2024-07-17

**Authors:** Haoxin Ren, Yining Li, Yi Yin, Sicheng Liu, Jingqi Zhang, Jingyu Zhang, Peilin Li, Zhe Wang, Peng Zhang

**Affiliations:** School of Space Science and Physics, Shandong University, Weihai 264209, China; haoxin-ren@mail.sdu.edu.cn (H.R.); yiningli@mail.sdu.edu.cn (Y.L.); yinyi@mail.sdu.edu.cn (Y.Y.); liusicheng@mail.sdu.edu.cn (S.L.); zhangjingqi@mail.sdu.edu.cn (J.Z.); zhangjingyu@mail.sdu.edu.cn (J.Z.); lipeilin1623@mail.sdu.edu.cn (P.L.); wangzhe2024@sdu.edu.cn (Z.W.)

**Keywords:** molybdenum, sodium molybdate, IR, Raman, vibrational mode, PPRA

## Abstract

Molybdenum (Mo) is a rare and important element extensively utilised in aerospace, radar communications, optoelectronic devices, and the military. This study proposes an environmentally friendly physical method based on photon–phonon resonance absorption for the separation of Mo from sodium molybdate (Na_2_MoO_4_). We examined the vibrational spectrum of Na_2_MoO_4_ using the CASTEP code, employing first-principles density functional theory. Through dynamic process analysis, we analysed the vibrational modes and assigned peaks corresponding to experimental infrared (IR) and Raman data. We focused on the vibrational modes associated with Mo and identified that the highest-intensity IR-active peak at 858 cm^−1^ corresponded to Mo–O bond asymmetric stretching. Therefore, we propose the use of a high-power terahertz laser at ~25 THz to facilitate the separation of Mo from Na_2_MoO_4_. Experimental investigations are expected in the future.

## 1. Introduction

Molybdenum (Mo) is a refractory metal with a body-centred cubic structure. It is widely used in aerospace, radar communication, equipment manufacturing, the nuclear industry, and other key engineering fields [1,2,3,4], owing to its high hardness, excellent thermal conductivity [5], electrical conductivity [6], and pressure resistance [7], and good corrosion resistance [6]. Moreover, it is an essential strategic resource in the development of science and technology [8] and has attracted much attention in recent years. Specifically, Mo can be used in missiles, nuclear energy materials, key components of turbines and fusion reactors, and corrosion-resistant coatings [9,10,11]. The compounds and alloy materials of Mo also have important applications [12]. For example, molybdenum disulfide can be used in the electronics industry [12], such as in memory applications [13], and molybdenum oxide can be used as a surface-enhanced Raman spectroscopy substrate [14].

At present, there are numerous methods for preparing Mo, including hydrogen reduction [15,16,17], thermal decomposition [18,19], and chemical vapour deposition [20]. Molybdates are important intermediate products in the traditional Mo production process [21,22]. There are primarily two methods for producing Mo from molybdate. First, molybdate is subjected to thermal decomposition to yield molybdenum oxide. Subsequently, the hydrogen reduction technique is applied to gradually convert the oxide into Mo through temperature adjustment [16]. In the reduction process, there are different proportioning oxides. Each of them requires different temperatures, resulting in inefficiencies. It is difficult to control combustion. The second approach involves directly heating the molybdate to facilitate its reaction with an active metal, resulting in the production of Mo [23]. It is necessary to control the salt-to-reactant ratio. And the finer the molybdenum powder outcome, the more byproducts there will be. Mo(CO)_6_ and molybdenum chloride are employed in the thermal decomposition method, which is applied in large-scale production by factories. However, the raw materials used in this process have stringent requirements. Special attention must be given to handling issues such as exhaust gas discharge and recycling treatment during their utilisation. Chemical vapour deposition involves the use of radio frequency-induced plasma, direct-current arc plasma, or microwave plasma [20]. This technique necessitates plasma equipment, leading to high equipment requirements and significantly increased production costs. Moreover, while nano-scale Mo powder can be prepared, the output rate is low, rendering it unsuitable for large-scale industrial production. Radio frequency-induced plasma and direct-current arc plasma technologies struggle to achieve a uniform temperature field, while microwave plasma can be disrupted by external reactants [20]. These methods necessitate high-temperature conditions for Mo synthesis, resulting in significant energy consumption. There is an urgent need for a novel, environmentally friendly technology for the large-scale extraction of Mo from ore with minimal pollution, high efficiency, and suitability for industrial production.

Numerous spectroscopic studies have focused on sodium molybdate (Na_2_MoO_4_) [24,25,26,27,28,29,30,31,32,33,34], a crucial and common molybdate. Mahadevan et al. found that ν_1_ and ν_2_ vibrational modes were absent in the infrared (IR) spectra of Na_2_MoO_4_, which indicated that the MoO_4_^2−^ ion retained $T_{d}$ symmetry in anhydrous Na_2_MoO_4_ [25]. In a related study, Raman spectroscopy measurements were conducted on polycrystalline Na_2_MoO_4_·2H_2_O and Na_2_MoO_4_ under hydrostatic pressure. The latter study monitored the stretching and bending vibrations of MoO_4_^2−^ [32]. Saraiva et al. provided evidence that the Mo–O bond lengths decreased in a high-temperature phase [30]. Clark and Doyle explored the IR spectra of 19 molybdates in terms of the site symmetry of the anionic group [35]. Abbas et al. explored the mechanical, thermodynamic, electronic, and optical properties of Na_2_MoO_4_ spinel through a density functional theory (DFT)-based full-potential augmented-plane-wave method [36].

In this study, we simulated the vibrational spectrum of Na_2_MoO_4_ and compared it with the experimental spectrum to accurately assign the Raman and IR peaks. Based on the IR-active peaks related to Mo–O, we propose a new method, namely photon–phonon resonance absorption (PPRA), to enhance the separation process of Mo from oxides [37,38].

## 2. Method

A primitive cell of the Na_2_MoO_4_ crystal contains two molecules. According to first-principles DFT simulations, we performed geometric optimisation and phonon calculations using the Cambridge Serial Total Energy Package (CASTEP 6.0) code [39]. Considering that electron density fluctuates substantially in the Na_2_MoO_4_ crystal, we employed the generalised gradient approximation of the revised Perdew–Burke–Ernzerhof (RPBE) exchange–correlation functional. The convergence tolerances of both the self-consistent field and ground energy were set to 1 × 10^−9^ eV/atom. The k-point mesh was 3 × 3 × 3, and the energy cutoff was set to 830 eV. The linear response approach was used to calculate IR and Raman intensities using norm-conserving pseudopotentials.

Based on the IR calculations, we analysed the dynamic processes of the vibrational modes at the gamma point. Consequently, we could attribute the peaks in the simulated IR and Raman spectra to their corresponding vibrations. This enabled us to assign the reported experimental peaks and identify the Mo-related vibrations with strong photon–phonon coupling. Finally, we selected the frequency for the terahertz laser that exhibited a high PPRA effect with the Na_2_MoO_4_ crystal.

## 3. Results and Discussion

**Figure 1:**
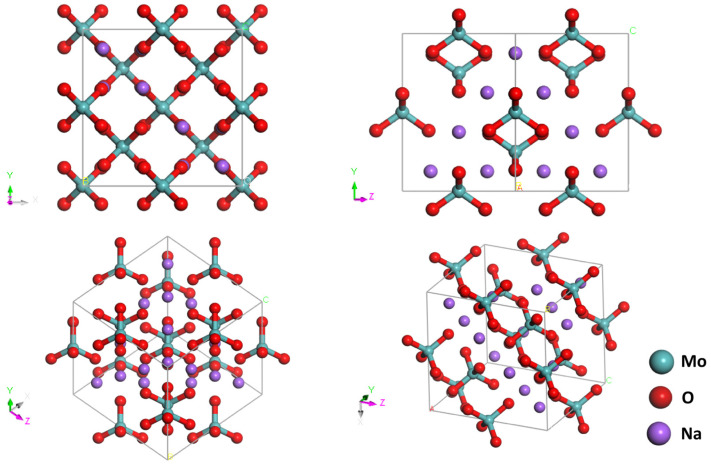
Conventional cell of Na_2_MoO_4_. Four views are presented to clarify the symmetry. There are four molecules in the conventional cell. A, B, and C stand for the three base vectors.

**Figure 2:**
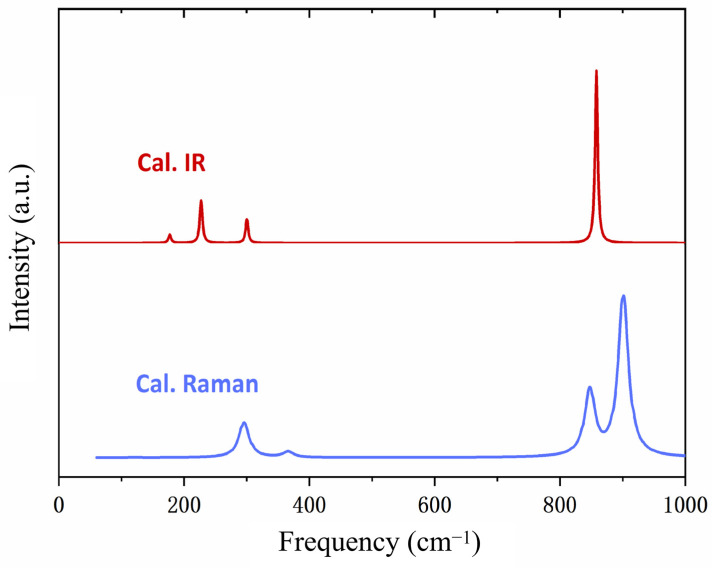
Simulated Raman scattering and IR absorption spectra. The main peaks in the IR spectrum are complementary to those in the Raman spectrum. The prominent peak in the IR spectrum is located at 858 cm^−1^.

The Na_2_MoO_4_ crystal features a spinel structure with the space group of *Fd3m* [24]. This structure consists of isolated tetrahedral MoO_4_^2−^ ionic groups. Figure 1 illustrates the conventional cell structure, which is face-centred and comprises eight formula units. To simulate the vibrational spectra, we used the minimum periodic structure, i.e., the primitive cell, for calculations. The primitive cell contains only two formula units, with lattice parameters set as a=b=c=6.537Å and α=β=γ=60°. Under the harmonic approximation, there are 14×3=42 vibrational modes, comprising 39 optical modes and 3 acoustic modes. We solely focused on the optical modes, as there is no resonance absorption between photons and acoustic phonons. According to group theory, the optical modes are distributed among the irreducible representations of the factor group *O_h_^7^*, expressed as A_1g_ + E_g_ + F_1g_ + 3F_2g_ + 2A_2u_ + 2E_u_ + 4F_1u_ + 2F_2u_ [32]. Subject to the selection rules, only the A_1g_, E_g_, and F_2g_ modes are Raman-active. Owing to the symmetric structure of Na_2_MoO_4_, changes in the dipole moment and polarisability of the primitive cell are mutually repulsive, resulting in a complete complementarity of IR-active and Raman-active modes. Figure 2 illustrates the simulated IR and Raman spectra. Based on DFT calculations, we can analyse the dynamic process of each vibrational mode. We classified all of the optical modes into four groups: rotation, translation, bending, and stretching, as listed in Table 1.

Table 1 compares the calculated wavenumbers with the experimental IR and Raman peaks. The last column presents a rough assignment of the 39 optical vibrational modes. In the lowest frequency band, there are 21 modes representing skeleton deformation vibrations, which are based on inter-molecular interactions. The intra-molecular bond angle vibrations (referred to as bending) exhibit high energy, while the inter-atomic stretching vibrations are located in the highest frequency band. The vibrational frequency can be simplified as that of a spring oscillator, given by $\omega =\sqrt{\frac{k}{m}}$. We identify 12 Raman-active modes. Among them, the modes at 120, 366, and 848 cm^−1^ exhibit triple degeneracy, and the mode at 296 cm^−1^ exhibits double degeneracy. There are also 12 IR-active modes, which consist of four triply degenerate modes at 138, 181, 285, and 834 cm^−1^, respectively. However, the other 12 modes exhibit neither IR nor Raman activity; these vibrational modes cannot be detected by IR or Raman spectrometry owing to selection rules. Each mode is unique; degenerate modes may vibrate in different directions or represent different combinations.

**Figure 3:**
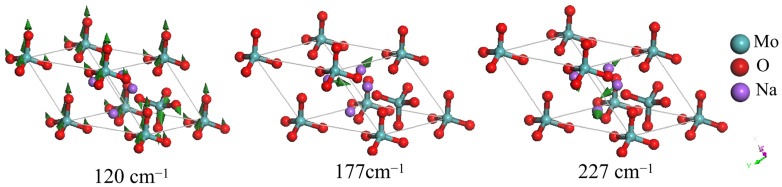
Three detectable vibrational modes at low frequencies. The mode at 120 cm^−1^ represents a relative movement of anion groups (Raman). The modes at 177 and 227 cm^−1^ are relative rotations (IR). The green arrows indicate the vibrational direction in proportion to the amplitude.

Busy and Keller observed a Raman peak at 116 cm^−1^, matching the 120 cm^−1^ peak in the present study, and defined it as a lattice vibration [24]. Through dynamic process analysis, we observe that when one MoO_4_^2−^ group vibrates in one direction, the neighbouring MoO_4_^2−^ groups vibrate in the opposite direction, resulting in three degenerate modes. Figure 3 depicts an example of these three modes at 120 cm^−1^. The eight anionic groups at the vertex angles represent one unit in a primitive cell. The three modes at 124 cm^−1^ represent relative rotations between the anionic group and Na^+^. Other modes below 284 cm^−1^ are also group rotations. We present two IR detection examples at 177 and 227 cm^−1^ in Figure 3. The mode at 284 cm^−1^ represents Na^+^ translation.

**Figure 4:**
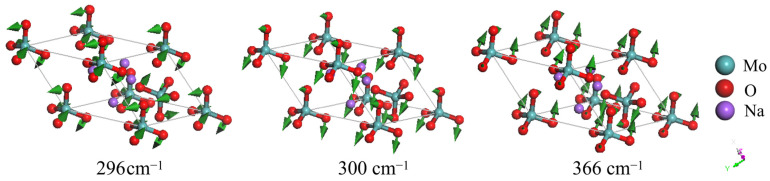
Three detectable vibrational modes in the bending band. They represent different combinations of intra-anionic bending vibrations. The modes at 296 and 366 cm^−1^ are Raman-active, while the mode at 300 cm^−1^ is IR-active. The green arrows indicate the vibrational direction in proportion to the amplitude.

We attribute the first 10 modes below 400 cm^−1^ to bending motions of the anionic groups because of the presence of clear deformations in the intra-anionic bond angles, as shown in Figure 4. In these modes, the oxygen atoms vibrate in different directions while the central Mo atom remains stationary. Chatterjee et al. attributed vibrational frequencies below 500 cm^−1^ to lattice vibrations [27]. In our study, first-principles calculations provide detailed insights into the dynamic processes of each vibrational mode, facilitating accurate assignments.

**Figure 5:**
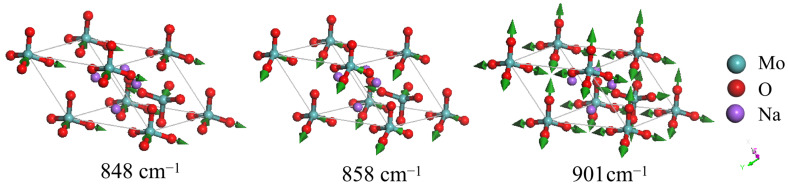
Three examples of detectable modes in the stretching band. The modes at 848 and 858 cm^−1^ represent Mo–O asymmetric stretching, while the mode at 901 cm^−1^ represents Mo–O symmetric stretching. The green arrows indicate the vibrational direction in proportion to the amplitude.

An energy gap exists between 366 and 848 cm^−1^. Given that the Mo–O bond is the strongest bond in molybdate, the Mo–O-related stretching modes are located in the highest frequency region. Mahadevan et al. observed a very strong peak at 816 cm^−1^ in the FTIR spectrum, along with a strong peak at 811 cm^−1^ and a weak peak at 849 cm^−1^ in the Raman spectrum [25]. Busy and Keller classified the 808 cm^−1^ lines of the Raman peak as ν_3_(f_2_) vibrations [24]. Saraiva et al. attributed the peak vibration pattern of 894 cm^−1^ (ν_1_) to stretching vibrations [30]. Additionally, Busy and Keller assigned the vibration mode corresponding to the peak of 892 cm^−1^ to symmetric stretching ν_1_(a_1_) vibration [24]. Chatterjee et al. assigned the peaks at 811, 838, and 895 to stretching vibrations [27]. In the present study, dynamic analysis shows that the eight calculated frequencies at wavenumbers 848, 858, 896, and 901 cm^−1^ represent different stretching vibrations. The three degenerate modes at 848 cm^−1^ (Raman-active) and three degenerate modes at 858 cm^−1^ (IR-active) correspond to Mo–O asymmetric stretching. The two modes at 896 and 901 cm^−1^ correspond to Mo–O symmetric stretching. The mode at 896 cm^−1^ is not detectable, while the strongest peak in the Raman spectrum is observed at 901 cm^−1^. Figure 5 illustrates three examples of detectable modes at 848, 858, and 901 cm^−1^, respectively. A detailed comparison of the vibrations at 858 and 901 cm^−1^ can be found in the Supplementary Information Files.

In summary, according to the calculation results, a sharp simulated IR peak at 858 cm^−1^ is shown in Figure 2, while the largest Raman peak is at 901 cm^−1^. These can be attributed to asymmetric and symmetric stretching, respectively. Considering that IR absorption is due to photon–phonon coupling, the experimental peaks around 830 cm^−1^ feature the most efficient photon–phonon absorption. Taking one vibrational mode as a harmonic oscillator, the phonon energy is $E=\left(n+\frac{1}{2}\right)\hbar \omega$. In the case of photon–phonon resonance absorption, if one photon energy is equal to ℏω, this vibration mode will obtain an energy-level transition with n plus one. Applying a high-power terahertz laser at this resonance frequency to a Na_2_MoO_4_ crystal will efficiently enhance the energy-level transitions of Mo–O vibrational-mode phonons. This may facilitate chemical bond breakage and improve Mo separation from Na_2_MoO_4_, offering a novel, pollution-free physical method for Mo separation.

The anionic group in molybdate, MoO_4_^2−^, is independent of the cation, and its vibrational spectrum remains unchanged regardless of the type of cation [35]. Consequently, the characteristic peaks of the Mo–O bond are very stable in IR/Raman spectra. Therefore, the analysis of Na_2_MoO_4_ crystals has implications for the study of other molybdate salts.

## 4. Conclusions

To assign the vibrational spectrum of molybdate, we use Na_2_MoO_4_ as a model to perform IR and Raman spectrum simulations. Experimental peaks are assigned according to the analysis of the dynamic processes of the vibrational modes. Owing to the symmetric structure of Na_2_MoO_4_, changes in the dipole moment and polarisability of the primitive cell are mutually repulsive. Consequently, the IR-active and Raman-active modes are complementary. The results show that there are 21 modes representing skeletal deformations in the low-frequency region. However, only two Raman peaks and two IR peaks can be detected, owing to selection rules and energy-level degeneracy. The vibrational energies of the 10 bending modes of MoO_4_^2−^ are relatively high, at ~300 cm^−1^. Two Raman peaks and one IR peak are detected in this region. Eight stretching vibration peaks exist in the energy band above 800 cm^−1^. These eight peaks consist of different symmetric or asymmetric Mo–O stretching combinations. Two strong Raman peaks occur with one prominent IR peak in this region. The simulations agree well with the experimental data. The results of first-principles calculations provide clear assignments for the experimental observations.

We focus on the Mo–O-related vibrational modes and confirm that the modes above 800 cm^−1^ correspond to O–Mo stretching vibrations. Among these, the significant IR peak represents Mo–O asymmetric stretching. The intensity of emergent light from Raman scattering is only $10^{-7}\sim 10^{-9}$ of the incident intensity. This means that photon–phonon resonance absorption is the most efficient method for photothermal conversion. We propose that the theoretical frequency of 858 cm^−1^ (when the experimental frequency is approximately 830 cm^−1^ or 25 THz) should be used as the terahertz laser frequency for radiation thermal conversion to aid in the separation of Mo from molybdate. The proposed PPRA method has potential applications in ice melting and gas hydrate mining [37,38,41]. However, verifying its potential is challenging owing to the requirement for terahertz-range free-electron laser equipment capable of providing a selected high-power laser frequency. Experimental investigations are expected in the future.

## Figures and Tables

**Table 1:** Comparisons between 39 calculated vibrational modes. The first column displays the wavenumbers (unit cm^−1^). The IR and Raman activities are labelled in the second column, followed by the experimental data in the next two columns. The corresponding vibrational mode assignment is marked in the last column.

Wavenumber	Activity	IR Exp.	Raman Exp.	Assignment
120	Raman		116 ^a^ 124 ^b^ 121 ^e^ 120 ^f^	MoO_4_^2−^ translation
120	Raman			MoO_4_^2−^ translation
120	Raman			MoO_4_^2−^ translation
124	Not active			Relative rotation
124	Not active			Relative rotation
124	Not active			Relative rotation
177	IR	177 ^b^ 177 ^f^		Relative rotation
177	IR			Relative rotation
177	IR			Relative rotation
192	Not active			MoO_4_^2−^ rotation
192	Not active			MoO_4_^2−^ rotation
192	Not active			MoO_4_^2−^ rotation
202	Not active			Relative rotation
202	Not active			Relative rotation
227	IR	230 ^b^ 290 ^d^ 230 ^e^ 230 ^f^		Relative rotation
227	IR			Relative rotation
227	IR			Relative rotation
256	Not active			Relative rotation
256	Not active			Relative rotation
256	Not active			Relative rotation
284	Not active			Na^+^ translation
296	Raman		303 ^a^ 311 ^b^ 330 ^c^ 319 ^c^ 317 ^c^ 305 ^e^ 305 ^f^	MoO_4_^2−^ bending
296	Raman			MoO_4_^2−^ bending
300	IR	325 ^a^ 317 ^b^ 313 ^d^ 317 ^f^		MoO_4_^2−^ bending
300	IR			MoO_4_^2−^ bending
300	IR			MoO_4_^2−^ bending
364	Not active			MoO_4_^2−^ bending
364	Not active			MoO_4_^2−^ bending
366	Raman		381 ^a^ 388 ^b^ 383 ^e^ 383 ^f^	MoO_4_^2−^ bending
366	Raman			MoO_4_^2−^ bending
366	Raman			MoO_4_^2−^ bending
848	Raman		808 ^a^ 849 ^b^ 845 ^c^ 823 ^c^ 841 ^c^ 811 ^e^ 811 ^f^	MoO_4_^2−^ stretching
848	Raman			MoO_4_^2−^ stretching
848	Raman			MoO_4_^2−^ stretching
858	IR	838 ^a^ 816 ^b^ 830 ^d^ 816 ^f^		MoO_4_^2−^ stretching
858	IR			MoO_4_^2−^ stretching
858	IR			MoO_4_^2−^ stretching
896	Not active			MoO_4_^2−^ stretching
901	Raman		892 ^a^ 897 ^b^ 898 ^c^ 891 ^c^ 894 ^f^	MoO_4_^2−^ stretching

^a^, Ref. [24]; ^b^, Ref. [25]; ^c^, Ref. [40]; ^d^, Ref. [35]; ^e^, Ref. [32]; ^f^, Ref. [30].

## Data Availability

Data are available from the authors on request.

## Supplementary Materials

The following supporting information can be downloaded at: https://www.mdpi.com/article/10.3390/molecules29143348/s1, Movie S1: The dynamic process of the vibrational mode at 858 cm^−1^; Movie S2: The dynamic process of the vibrational mode at 901 cm^−1^.
